# Association between Dietary Patterns and Metabolic Syndrome and Modification Effect of Altitude: A Cohort Study of Tibetan Adults in China

**DOI:** 10.3390/nu15092226

**Published:** 2023-05-08

**Authors:** Haijing Wang, Yanxiang Wang, Zumin Shi, Lei Zhao, Wenxiu Jian, Ke Li, Ruihua Xu, Yan Wu, Fei Xu, Youfa Wang, Wen Peng

**Affiliations:** 1 Nutrition and Health Promotion Center, Department of Public Health, Medical College, Qinghai University, Xining 810008, China; 2 Human Nutrition Department, College of Health Sciences, QU Health, Qatar University, Doha 2713, Qatar; 3 Global Health Institute, School of Public Health, Xi’an Jiaotong University, Xi’an 710061, China; 4 Department of Epidemiology, School of Public Health, Nanjing Medical University, Nanjing 211166, China; 5 Non-Communicable Disease Prevention and Control, Nanjing Municipal Center for Disease Control and Prevention, Nanjing 211166, China; 6 Qinghai Provincial Key Laboratory of Prevention and Control of Glucolipid Metabolic Diseases with Traditional Chinese Medicine, Xining 810008, China

**Keywords:** blood pressure, blood glucose, dietary pattern, high altitude, lipids, metabolic syndrome, obesity, Tibetans

## Abstract

Little is known about the longitudinal association between dietary patterns (DPs) and metabolic disorders in people living at high altitude areas, such as Tibetans. We constructed the first open cohort, with 1832 Tibetans, and collected data in 2018 and in 2022. The metabolic syndrome (MetS) prevalence was 30.1% (32.3% in men and 28.3% in women). Three different DPs were identified: modern DP (pulses, poultry, offal, and processed meat), urban DP (vegetables, refined grain, beef/mutton, and eggs), and pastoral DP (Tibetan cheese, tsamba, butter/milk tea, and desserts). Participants within the third tertile of the urban DP had a 3.42-fold (95% CI 1.65–7.10) higher risk of MetS than those with the first tertile. Modern DP was positively associated with elevated blood pressure (BP) and elevated triglycerides (TAG), while it was inversely associated with low HDL-C. The urban DP was associated with a higher risk of low HDL-C, but a lower risk of impaired fasting blood glucose (FBG). The pastoral DP was a risk factor for impaired FBG, but protective for central obesity and elevated BP. Associations of modern DP with elevated BP, and pastoral DP with low HDL-C, were modified by altitude. In conclusion, among Tibetan adults, DPs were associated with MetS and its components, and the associations were modified by altitude among Tibetans.

## 1. Introduction

Metabolic syndrome (MetS) is a cluster of metabolic abnormalities that include central obesity, elevated blood pressure (BP), elevated triglycerides (TAG), low high-density lipoprotein cholesterol (HDL-C), and impaired fasting blood glucose (FBG) [1]. MetS has direct effects on cardiovascular and cancer risks, and accounts for the largest burden of non-communicable diseases worldwide [2,3]. The global prevalence of MetS has been reported to be about 25%. The prevalence of MetS has been increasing in China over the past three decades, especially among urban residents [1,4]. In particular, obesity and diabetes have increased substantially, putting a huge burden on China’s health-care system [5,6]. A meta-analysis reported that the pooled prevalence of MetS in China was 24.5%, but it varied greatly among ethnic groups, with a reported prevalence of 6.17% among Tibetans [7,8]. Our recent study showed that the prevalence of MetS (men 30.1% and women 32.1%) in the Tibetan population transitioning from nomadic (very high altitude) to urban and suburban areas (high altitude) was similar to the national rate [9].

MetS is a multifactorial disease that is influenced by the interactions of genetic, environmental, and lifestyle factors, and among these, the modifiable factors among Tibetans have attracted considerable interest, especially dietary habits. Diet is an important modifiable factor that can affect MetS risk. The western dietary pattern (DP), characterized by a high consumption of unhealthy elements, for example, sweetened drinks, snacks, and desserts, has been shown to be associated with the risk of developing MetS and its components [9,10]. On the contrary, Mediterranean-style dietary approaches to stop hypertension (DASH), i.e., low-carbohydrate and low-fat diets can ameliorate MetS [11,12]. The environment has also been suggested to potentially influence metabolic diseases, and oxidative stress is closely associated with MetS [13]. Nutritional stress and high altitude both influence oxygen availability and promote oxidative stress [14,15]. Although high altitude has been proven in studies to be a protective factor for MetS and its components [16,17], the presence of an interaction between food and altitude deserves further investigation.

Tibetans live on the world’s highest plateau, and the unique geographical locations of their residences have contributed to the distinct and traditional customs associated with their diet, which mainly includes tsamba, yak milk, Tibetan noodles, butter tea, and Qing cha [18]. Due to the extreme altitude of pure pastoral areas, the local traditional diet of the settled Tibetan population has been livestock-based. A previous cross-sectional study in Lhasa found that tsamba, butter tea, and Qing cha were inversely associated with MetS [19]. Cross-sectional studies have shown that, due to drastic social and economic changes, traditional pastoral dietary habits have changed, and the urban DP has been identified as a risk factor for MetS [9,20]. However, no cohort study was found that examined the associations between DPs and MetS among the Tibetan population. Further, the role of altitude has not been explored.

This study investigated the effects of DPs on MetS and its components in an open cohort of Tibetan adults and examined the modification effects of altitude on the associations.

## 2. Materials and Methods

### 2.1. The Participants and Study Design

In this community-based, open cohort study, data were collected twice in 2018 and 2022 [9,21]. The participants came from two Tibetan communities in the Golmud suburbs (2800 m above sea level) in which, since 2007, the community members had gradually settled or semi-settled from pure pastoral areas (>4000 m). The geographical features and background of the communities have been previously described [22]. In short, some community members had totally abandoned pasture life and almost permanently resided in an urban environment (urbanized). Other community members had maintained connections to their pastoral links, either by owning livestock that are cared for by others or by periodically moving between urban and pastoral lifestyles (semi-urbanized).

**Figure 1:**
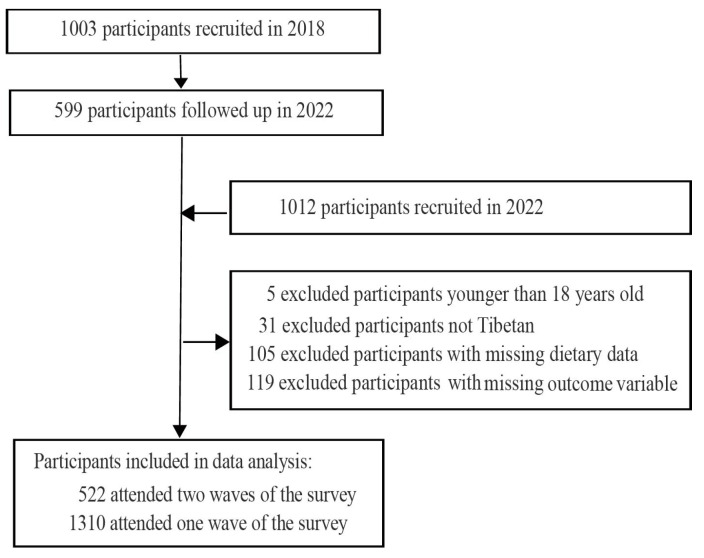
The flowchart of participants.

In this study, 1832 participants completed at least one wave of the 2 surveys that included baseline and follow-up data collection, and 522 of the participants completed two waves of the surveys (Figure 1). The survey enrolled 1003 participants who voluntarily registered for the check-up program from November 2018 to December 2018, and 1611 participants from December 2021 to May 2022. We excluded 260 subjects who were non-Tibetans (*n* = 5), under the age of 18 (*n* = 31), failed to complete the dietary survey (*n* = 105), or had missing outcome variables (*n* = 119). Data were collected using questionnaires, anthropometric measurements, and biomarker tests in 2018 and in 2022, accomplished by well-trained investigators and local medical workers.

All participants gave their informed consent for inclusion before they participated in the study. The study was conducted in accordance with the Declaration of Helsinki, and the protocol was approved by the Ethics Committee of the Medical College, Qinghai University (2017-34, 2021-15).

### 2.2. Outcome Variable: The Criteria for MetS and Its Components

The anthropometric and biomarker measurements of participants have been previously detailed [9]. A modified definition of MetS was used [23]. Participants were considered to have MetS if they met three or more of the following criteria: (1) central obesity, i.e., waist circumference ≥90 cm for men or ≥80 cm for women; (2) elevated BP, i.e., systolic BP ≥ 130 mmHg, diastolic BP ≥ 85 mmHg, or self-reported hypertension; (3) low HDL-C, i.e., HDL-C < 1.03 mmol/L for men or <1.30 mmol/L for women, or using lipid-lowering drugs; (4) elevated TAG, i.e., TAG ≥ 1.7 mmol/l, or using lipid-lowing drugs; (5) impaired FBG, i.e., FBG ≥ 5.6 mmol/L or self-reported diabetes mellitus.

### 2.3. Exposure Variable: Dietary Assessment and Dietary Patterns

Dietary intake information was collected using a revised food frequency questionnaire (FFQ) for assessing self-reported food intake over the previous year. This FFQ was constructed based on similarity of nutrition and local dietary culture, and the details have been previously described [9]. Simply, the FFQ consisted of 27 food groups (varying from refined grains to special Tibetan food, e.g., tsama), and the consumption frequency of each food group in the previous year was assessed on a daily, weekly, monthly, and yearly basis. In the DP analysis, to gain full advantage of the food information, we excluded the mushroom food group which have has a lot of missing in 2022, and further converted all food consumption to frequencies per month.

DPs were identified using factor analysis based on a correlation matrix of each variable (principal-component factors method with varimax rotation). The Kaiser–Meyer–Olkin (KMO) measure of sampling adequacy (KMO = 0.771) and Bartlett’s test of sphericity (*p* < 0.001) indicated that the data were suitable for factor analysis. To determine the number of factors to retain, we considered eigenvalues, scree plots, and interpretability (Figure S1). Absolute factor loading after three factors rotated higher than 0.30 was selected as the cut-off value for identifying food groups that strongly contributed to a particular DP. Finally, three DPs were identified, and the cumulative variance explained 28.2% of the total variance. Then, the DP scores (factor scores) were calculated, and each DP was divided into three tertiles (T1–T3). T1 was the low score group, T2 and T3 were the medium and high score groups, respectively.

### 2.4. Covariates

A structured questionnaire was used to collect data including sociodemographic characteristics, disease history, and lifestyle factors. Furthermore, to evaluate the potential influence of altitude, we collected the time participants had resided in a pasturing area (>4000 m) (referred to as residence time). Participants were settled in the community before the investigation. Altitude was determined by the participant’s annual residence time in a pasturing area. Participants who lived in pasturing area for more than 6 months per year were classified as living at very high altitude (>4000 m), while those who lived in pasturing areas less than or equal to 6 months per year were classified as living at high altitude (2800 m).

In our analysis, potential confounders were selected by directed acyclic graphs (Figure S2) [24]. The following factors were included: sex, age [25], marital status (unmarried/widowed/divorced/separated, or married), education level (no schooling, <6 years of schooling, or ≥6 years of schooling), insurance (rural/no insurance, or urban insurance), household income (CNY < 20,000, CNY 20,000–100,000, or CNY ≥ 100,000), and self-reported physical activity (low, median, or high). Smoking status was categorized into never smoking, former smoker, <5 cigarettes/d (current), or ≥5 cigarettes/d (current). Alcohol drinking was categorized into never, abstinence, <40 g/week, or ≥40 g/week.

### 2.5. Statistical Analyses

Descriptive statistics were calculated using the mean ± standard deviation (SD) for continuous variables or *n* (%) for categorical variables. Student’s *t*-test or ANOVA was used to compare means; χ^2^ test was used to compare percentages. A mixed-effect logistic regression analysis was used to evaluate the associations of the three tertiles of DP scores with MetS and its components. The unadjusted and adjusted models included a random intercept to account for intra-subject variability. The results of the stationary effect were presented as odds ratios (ORs) values and 95% confidence intervals (CIs).

Five models were fit: Crude was an unadjusted model; Model 1 was adjusted for age and sex; Model 2 was further adjusted for marital, education, insurance, household income, smoking, alcohol drinking, and physical activity; Model 3 was further adjusted for altitude; Model 4 (sensitivity analysis) was the same as Model 3, but only included those who had both baseline and follow-up data. The linear trend was conducted to test the associations between DPs and MetS and its components, in which DPs were modeled as a continuous variable by using the median score of each tertile across the DP tertiles. We also presented an interaction and subgroups analysis between the three tertiles of DP scores and living altitude among participants who completed two waves of surveys.

Statistical analyses and graphic plotting were conducted using Stata version 17.0 and R version 4.2.1. OR values and 95% CI for T3 of DPs were visualized using ggplot2 [26], and the scale_x_log10 function was used. All *p*-values were 2-sided, and statistical significance was defined as *p* < 0.05. The multiple comparison adjustments were not used since this is an exploratory study based on observational data [27].

## 3. Results

### 3.1. Metabolic Syndrome and Dietary Patterns

In 2018 and in 2022, a total of 1832 participants were surveyed in our study, with 522 participants completing two waves of surveys and 1310 participants completing one wave of surveys (Figure 1). Most of the participants (80.7%) lived at high altitude, and the mean age was 43.13 ± 14.37 years old. The prevalence of MetS was 30.1% (men 32.3% and women 28.3%). Participants with MetS were significantly older, married, had a lower education level, and were engaged in less physical activity (Table S1). Changes in the MetS components were discovered in the two waves of surveys. Low HDL-C (86.7%) and central obesity (55.9%) were the leading abnormalities in 2018; however, in 2022, elevated BP (41.3%) was added as the second most common component of MetS in 2022.

Three different DPs were identified by factor analysis (Table 1). The modern DP was characterized by frequent consumption of pulses, whole grains, fresh fruit, nuts and seeds, different kinds of meat (i.e., offal, poultry and pork), and processed foods (i.e., processed vegetables and meat, sweetened drinks, and salty snacks). The urban DP featured more frequent intakes of refined grains, beef and mutton, eggs, and all kinds of vegetables (e.g., dark and light vegetables, etc.). The pastoral DP was characterized by frequent consumption of desserts and Tibetan special diets (e.g., Tibetan cheese, tsamba (roasted Tibetan barley), butter/milk tea, and whole-fat dairy).

### 3.2. Characteristics of the Participants among Different Dietary Patterns

Illustration of the demographic and lifestyle characteristics of 1832 participants who completed the first wave of the surveys based on three tertiles of three DP scores (Table 2). Participants with high modern DP scores (T3) were aged 39.28 ± 14.22 years old and had a median household income between CNY 20,000–100,000. Participants with high urban DP scores (T3) were engaged in light physical activity, smoking, and drinking. As a unique DP of the Tibetan population, participants with high pastoral DP scores (T3) were more likely to be married, have a low education level, with rural/no insurance, and live at very high altitudes.

### 3.3. Prospective Associations among Dietary Patterns Tertile Scores with MetS and Its Components

The results of the mixed-effect models showed associations among the three tertiles of three DPs with MetS and its components in Tibetan adults (Table 3). In Model 3, after adjusting for sociodemographic and lifestyle factors, as well as altitude, a higher score of urban DP score increased the risk of MetS, and the ORs (95% CI) for the T1, T2, and T3 were 1.00, 1.20 (0.79, 1.83), and 2.25 (1.36, 3.73), respectively. A similar positive association was observed in the sensitive analysis (Model 4). For central obesity, the higher score of pastoral DP decreased the risk after adjusting sociodemographic and lifestyle factors in Model 2, and the ORs (95% CI) were 1.00, 0.86 (0.54, 1.35), and 0.57 (0.35, 0.92), respectively. For elevated BP, the higher score of the modern DP was shown as a risk factor. However, the opposite relationship was observed in the pastoral pattern (Model 2). For low HDL-C, the higher score of the modern DP was associated with decreasing disease risk, and the ORs (95% CI) in Model 4 were 1.00, 0.82 (0.58, 1.17), and 0.60 (0.41, 0.87), respectively. In contrast, the higher score of the urban DP was associated with an increased risk of low HDL-C, and the ORs (95% CI) were 1.00, 1.42 (1.00, 2.01), and 2.47 (1.66, 3.69), respectively. For elevated TAG, the higher score of the modern DP showed an association with increased risk in Model 2, and the ORs (95% CI) were 1.00, 0.94 (0.63, 1.40), and 1.50 (1.00, 2.26), respectively. For impaired FBG, the higher score of urban DP was a protective factor in Model 2, and the ORs (95% CI) were 1.00, 0.95 (0.63, 1.43), and 0.49 (0.29, 0.81), respectively. However, after adjusting for altitude in Model 3, a higher pastoral DP score increased the risk of impaired FBG.

**Figure 2:**
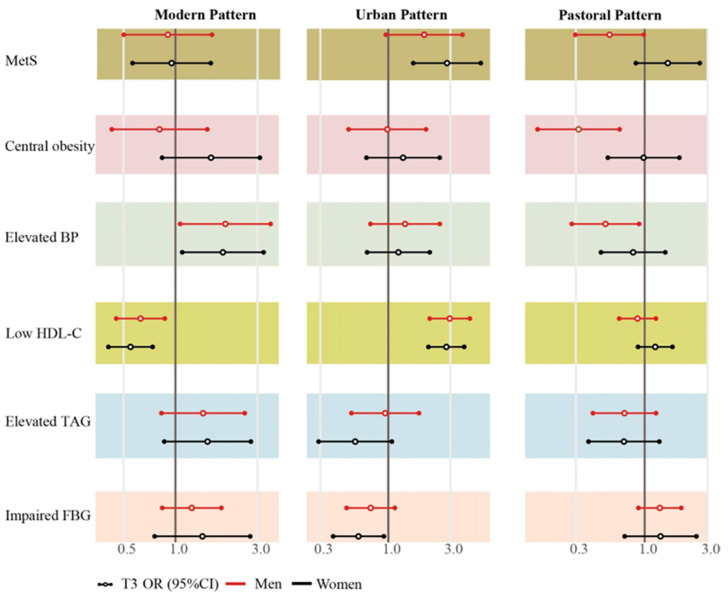
Associations between tertiles of three DP scores with MetS and its components by gender among Tibetans in China. Left panel shows modern DP. Middle panel shows urban DP. Right panel shows pastoral DP. DP scores are equally divided into thirds, and T means tertile (T1–T3). Only T3 was illustrated here. Model was adjusted for age, marital status, education, insurance status, household income, smoking status, alcohol consumption, and physical activity. The detailed information is in Table S2.

Considering gender difference, the urban DP was inversely associated with MetS only in women, and the ORs (95% CI) were 1.00, 1.28 (0.71, 2.30), and 2.91 (1.42, 5.99), respectively, whereas the association of modern DP with MetS and its components was consistent for both men and women. The intriguing results were found in the pastoral DP, i.e., a high score of pastoral DP (T3) was a protective factor for MetS, central obesity, and elevated BP in men (Figure 2, Table S2). These findings revealed that the three DPs had different effects on men’s and women’s metabolic health outcomes.

### 3.4. The Effect of Dietary Patterns Was Modified by Altitude

**Figure 3:**
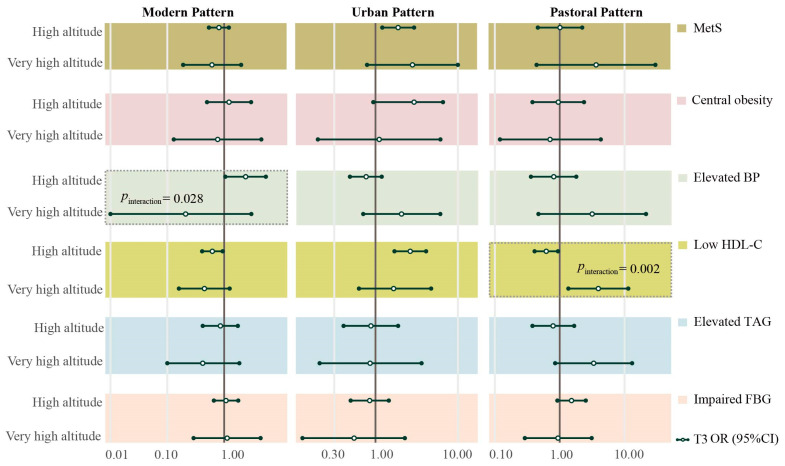
Subgroup analyses of the associations between tertiles of three major DP scores and MetS and its components among participants who attended two waves of the survey (*n* = 522). Left panel shows modern DP. Middle panel shows urban DP. Right panel shows pastoral DP. DP scores are equally divided into thirds, and T means tertile (T1–T3). Only T3 was illustrated here. Model adjusted for age, sex, marital status, education, insurance status, household income, smoking status, alcohol consumption, and physical activity. The detailed information is in Table S3.

Interactions between altitude and DPs in relation to components of MetS were identified in the fully controlled Model 4 which should not be ignored, although the same results were not observed in Model 3. Significant interaction was identified between modern DP (*p*_interaction_ = 0.028) and altitude, and between pastoral DP (*p*_interaction_ = 0.002) and altitude in relation to elevated BP and low HDL-C, respectively (Figure 3 and Table S3). Participants only living at high altitude with a high score (T3) were most likely to be diagnosed with elevated BP as compared with those with a low score (T1), the OR (95% CI) was 2.39 (1.05, 5.42). Pastoral DP was identified as a protective factor at high altitude for low HDL-C; and ORs (95% CI) for T1, T2, and T3 were 1.00, 0.94 (0.63, 1.40), and 0.62 (0.41, 0.94), respectively. However, it was reversed at very high altitude, and ORs (95% CI) were 1.00, 3.32 (1.13, 9.76), and 3.94 (1.36, 11.41), respectively.

## 4. Discussion

To the best of our knowledge, this is the first cohort study of Tibetan adults that examined the associations between DPs and metabolic risk. Further, for the first time, the modification effects of high altitude on the associations were also explored. Three DPs were identified, with urban DP being a risk factor for MetS. For the components, modern DP increased the risk of elevated BP and elevated TAG, but decreased the risk of low HDL-C. The urban DP score showed a strong positive association with low HDL-C and an inverse association with impaired FBG. As a unique dietary pattern of Tibetan adults, pastoral DP was identified as a protective factor for central obesity and elevated BP, while it was a risk factor for impaired FBG. Modern DP had the most consistent gender effect, and the protective effects of pastoral DP on MetS, central obesity, and elevated BP were more pronounced in men.

Tibetans in the process of urbanization are in a state of nutritional transition, which has been linked to increase the risk of MetS. The prevalence of MetS (30.1%) in the urbanized and semi-urbanized Tibetans adults was higher than that in the native pastoral setting (ranging from 3.6% to 17.3%) [8,9,28,29] and similar to the urbanized Jiarong Tibetan population in the Aba area (37.6%) [20], implying that changes in living environment brought new challenges. Urbanization weakens the consumption of traditional foods and increases the consumption of industrial foods, especially among young people. Our results also confirmed that participants with higher modern DP scores were younger, while the elderly were more likely to follow the traditional DP. Most studies have deemed the risk of noncommunicable diseases increased, especially obesity and other metabolic disorders, during the urbanization process [30,31]. Urbanized Tibetans have easier access to the diversification of food; however, this change may increase the prevalence of MetS.

The modern DP contained a broader range of nutrients, with an increased consumption of unhealthy foods such as offal and processed foods, and healthy foods such as pulses, whole grains, and fresh fruit, which was different from the previously identified western DP [9]. The sample size for generating the DPs was larger than that of the previous study, and the Tibetans adopted more diversified food group from the urban environment. The modern DP seems to be a balanced diet; however, the negative effects outweigh the benefits of healthy foods. It has been shown that ultra-processed food increases the risk of obesity, diabetes, and hypertension among Canadian adults [32]. Processed foods, including snacks with increased sodium levels, have also been associated with increased BP [33]. One study showed that high intake of whole-grain foods could significantly reduce BP [34]; however, less than 4% of the participants had daily intakes of whole grains. Hypertension has been linked to an increased risk of cardiovascular disease, particularly in a younger population [35]. Indeed, the modern DP is mainly adopted by young people who are more confident about their physical health, and thus they are more likely to disregard a healthy diet. Saturated fatty acids (SFA) account for 45–70% of the fatty acids in the offal fat [36]. Although the relationship between SFA intake and hypertension is still being debated, SFAs are generally thought to be a risk factor for elevated BP [37,38]. Meanwhile, a higher SFA intake was associated with a higher HDL-C level, indicating a protective factor for low HDL-C levels [39]. However, intake of monounsaturated fatty acids, such as olive oil, have been shown to elevated the postprandial plasma TAG concentration as compared with an SFA enriched diet [40]. With the availability of food resources, young people are increasingly preferring high-fat, high-salt foods. The rising burden of noncommunicable diseases and their link to lifestyle is a global concern, and targeted dietary education should be implemented to promote a more balanced diet.

The urban DP is distinguished by higher carbohydrate (refined grains) and choline (beef, mutton, and eggs) levels, and dietary choline, which are precursors of trimethylamine N-oxide (TMAO), may play a role in MetS and its components. The evidence for a positive association between eggs, red meat, and refined carbohydrate consumption and MetS has been observed in different populations [41,42,43,44]. It is interesting to note that the urban DP was a risk factor for low HDL-C, whereas the modern DP was a protective factor. The substitution of SFAs with carbohydrates, particularly refined carbohydrates, has been associated with lower HDL-C, indicating that refined carbohydrates play a vital role in this shift [39,45]. Elevated plasma TMAO concentrations have also shown a specific metabolic pattern characterized by low HDL-C [46]. TMAO concentrations also explain, to some extent, the risk effect on low HDL-C. Changes in the levels of the sex hormone estradiol, as well as physiological changes in women during middle age, have been linked to metabolic changes and fat gain [47]. This may help to explain why the urban DP has significantly increased the risk of MetS in women, and the hormone levels should be investigated further. Although vegetables were included in the urban DP, the cooking method, including heavy oil and salt, may have an offset effect against their healthy impacts. The urban DP increases the local people’s food resources, such as vegetables and refined carbohydrate, and it may also introduce new and emerging risks in this population.

The pastoral DP as a traditional DP is dominated by dairy products and prevents several components of MetS. Some Tibetan habitual foods have been identified as protective factors for MetS [19], for example, tsamba is one of the most important staple food crops consumed by Tibetans, which is made of roasted Tibetan barley rich in β-glucan [48,49]. A randomized and double-blind study showed that high levels of β-glucan may have prevented visceral fat obesity [50]. Furthermore, dairy products rich in phosphorus and calcium may have contributed to the beneficial associations with BP [51]. The protective effect of this DP was significant in men, implying that hormone levels might play a role [52]. This finding also highlights the importance of taking gender differences into account when developing appropriate dietary strategies. Ethnic-specific dietary guidelines should include health-promoting components derived from traditional DP. However, the extension to other populations remains unknown.

The modifiable effect of altitude showed that a high score of pastoral diet was a risk factor for lower HDL-C at very high altitudes, but a protective factor at high altitudes. We hypothesize that hypoxia might affect metabolism, resulting in different outcomes for the same DP. Plasma TMAO concentrations increase with increased consumption of milk and other dairy products, and TMAO concentration has been inversely correlated with HDL-C [53]. A high-fat diet impairs mitochondrial oxygen uptake into host enterocytes, allowing facultative anaerobes such as the pathobiont *Escherichia Coli* to take over, resulting in an increase in the amount of choline catabolized into the precursor for TMAO [54]. The relative abundance of anaerobic bacteria has been reported to be significantly increased in the hypoxic groups [55]. The production of TMAO might be increased by combining hypoxia and microbial pathways, which in turn, lower HDL-C. These findings indicated that the urban environment at a lower altitude as compared with a pasturing area was beneficial to health at a certain extent, especially in an older population. Further studies with larger sample sizes are needed to confirm our findings.

Understanding the influence of DPs on MetS and its components is crucial for developing public health interventions. The high prevalence of MetS is a threat to the long-term health of the Tibetan population with a potentially huge burden on local public health systems. Identifying risk factors for MetS would assist in the development of health promotion strategies to reduce morbidity and mortality in this vulnerable population. The Healthy China 2030 national strategy provides a historical opportunity to develop comprehensive strategies for the wellbeing of local Tibetans. Additionally, our study found interaction effects between altitude and DPs in relation to components of MetS in Tibetan adults, which to some extent fills the gap in the field of public health.

This study has several limitations. Firstly, dietary data collected using FFQ were only based on the consumption frequency without portion size and may have inaccurate measurements. However, research has indicated consumption frequency was more important than portion size for interpersonal variation [56]. Because of differences in health literacy, it is also likely there was recall bias when the frequency of food consumption in the past year was investigated. Secondly, external validity should be interpreted with caution, due to the particularity of the survey population. Thirdly, the limited sample of follow-up data should be expanded further to increase the robustness of the observed interactions. Nevertheless, no other cohort studies on chronic diseases in Tibetan adults were found by a previous systematic review [48], and therefore, the value and public health implications of this study are quite significant. For further research, the regulation of oxygen availability at different altitudes for the same DP is still unknown. Thus, research is still needed to examine the potential mechanisms, such as the gut microbiome–host metabolism axis.

## 5. Conclusions

In conclusion, three identified DPs are associated with MetS and its components. The modern DP was popular among young people, and its unhealthy elements may have elevated BP. The negative impact of the urban DP on MetS was further strengthened, and the protective effect of the pastoral DP among Tibetans should be given attention. Furthermore, the interaction of altitude and the pastoral DP relative to HDL-C was discovered for the first time. Public health efforts to promote cultural-adapted healthy eating among the target Tibetan adults are needed, and this study provides some evidence for tailored interventions.

## Figures and Tables

**Table 1:** Three major DPs identified among subjects in urbanized and semi-urbanized settled Tibetan communities ^1,2^.

Food Groups	Modern Pattern	Urban Pattern	Pastoral Pattern
	Factor 1	Factor 2	Factor 3
Pulses	**0.60**	0.06	−0.01
Poultry	**0.60**	0.05	0.00
Whole grains	**0.54**	0.08	−0.09
Offal	**0.53**	−0.07	0.06
Processed meat	**0.51**	0.10	−0.06
Fresh fruits	**0.50**	0.13	−0.03
Processed vegetables	**0.50**	−0.01	−0.04
Nut and seeds	**0.43**	0.20	0.08
Pork	**0.39**	0.14	−0.15
Sugar-sweetened beverages	**0.39**	0.27	−0.04
Salty snacks	**0.32**	0.24	0.05
Dark vegetables	0.13	**0.64**	−0.15
Light vegetables	0.13	**0.61**	−0.10
Refined grains	−0.12	**0.55**	−0.00
Tubes and roots	0.21	**0.54**	−0.09
Onion and spring onion	0.14	**0.49**	−0.15
Beef and mutton	−0.27	**0.45**	0.24
Eggs	0.20	**0.30**	−0.03
Tibetan cheese	−0.04	−0.08	**0.74**
Tsamba	−0.01	−0.21	**0.71**
Butter tea and milk tea	−0.06	0.27	**0.48**
Desserts	0.21	0.22	**0.41**
Whole-fat dairy	0.02	0.28	**0.32**
Fried foods	0.29	0.02	0.16
Seafood	0.21	0.07	−0.04
Non-caloric drink	−0.08	0.24	−0.28
**Variances explained (%)**	**11.68**	**9.47**	**7.02**
**Cumulative variance explained (%)**	**11.68**	**21.15**	**28.17**

^1^ Extraction method: principal component analysis with varimax rotation. Tables in bold indicate absolute factor loadings are higher than 0.30 and considered to belong to the corresponding dimension in the column. Red color: positive scores; blue color: negative scores; darker colors indicate higher DP scores. ^2^ Tibetan adults who attended at least one wave in 2018 and 2022 were included (*n* = 2354 person-time).

**Table 2:** Demographic and lifestyle characteristics of participants by tertiles of major DP scores of Tibetans in China (*n* = 1832) ^1^.

	Modern Pattern				Urban Pattern				Pastoral Pattern			
	T1	T2	T3	*p*	T1	T2	T3	*p*	T1	T2	T3	*p*
Survey year (%)				<0.001				<0.001				0.082
2018	393 (61.70)	320 (50.96)	232 (40.92)		189 (33.16)	261 (43.94)	495 (74.10)		292 (47.95)	333 (53.97)	320 (52.81)	
2022	244 (38.30)	308 (49.04)	335 (59.08)		381 (66.84)	333 (56.06)	173 (25.90)		317 (52.05)	284 (46.03)	286 (47.19)	
Age(years)	46.43 ± 13.51	43.25 ± 14.51	39.28 ± 14.22	<0.001	46.02 ± 15.34	43.37 ± 13.74	40.45 ± 13.54	<0.001	39.44 ± 14.90	44.92 ± 14.01	45.01 ± 13.45	<0.001
Sex (%)				0.362				0.404				0.163
Men	304 (47.72)	275 (43.79)	263 (46.38)		253 (44.39)	286 (48.15)	303 (45.36)		299 (49.10)	273 (44.25)	270 (44.55)	
Women	333 (52.28)	353 (56.21)	304 (53.62)		317 (55.61)	308 (51.85)	365 (54.64)		310 (50.90)	344 (55.75)	336 (55.45)	
Marital (%)				<0.001				0.105				<0.001
Unmarried/widowed/divorced/separated	53 (8.33)	87 (13.88)	111 (19.61)		90 (15.82)	83 (14.02)	78 (11.68)		128 (21.12)	65 (10.53)	58 (9.57)	
Married	583 (91.67)	540 (86.12)	455 (80.39)		479 (84.18)	509 (85.98)	590 (88.32)		478 (78.88)	552 (89.47)	548 (90.43)	
Education (%)				<0.001				<0.001				<0.001
No schooling	513 (81.56)	454 (72.52)	353 (62.70)		456 (80.28)	436 (74.02)	428 (64.75)		355 (58.68)	479 (78.14)	486 (81.00)	
<6 years of schooling	60 (9.54)	54 (8.63)	47 (8.35)		42 (7.39)	47 (7.98)	72 (10.89)		52 (8.60)	55 (8.97)	54 (9.00)	
≥6 years of schooling	56 (8.90)	118 (18.85)	163 (28.95)		70 (12.32)	106 (18.00)	161 (24.36)		198 (32.73)	79 (12.89)	60 (10.00)	
Insurance (%)				<0.001				<0.001				0.006
Urban insurance	234 (37.20)	213 (34.03)	277 (49.20)		127 (22.36)	180 (30.56)	417 (63.09)		271 (44.79)	237 (38.66)	216 (36.00)	
Rural/No insurance	395 (62.80)	413 (65.97)	286 (50.80)		441 (77.64)	409 (69.44)	244 (36.91)		334 (55.21)	376 (61.34)	384 (64.00)	
Household income (Yuan, %)				0.001				0.001				0.105
<20,000	165 (26.79)	160 (26.40)	93 (17.13)		139 (25.36)	138 (24.04)	141 (21.93)		137 (23.46)	130 (21.89)	151 (25.72)	
20,000~100,000	378 (61.36)	374 (61.72)	371 (68.32)		363 (66.24)	365 (63.59)	395 (61.43)		358 (61.30)	395 (66.50)	370 (63.03)	
100,000~	73 (11.85)	72 (11.88)	79 (14.55)		46 (8.39)	71 (12.37)	107 (16.64)		89 (15.24)	69 (11.62)	66 (11.24)	
Smoking (%)				0.073				<0.001				<0.001
Never	502 (79.94)	503 (80.35)	429 (76.20)		474 (83.60)	484 (82.17)	476 (72.01)		433 (71.69)	507 (82.71)	494 (82.33)	
Former smoker (%)	41 (6.53)	40 (6.39)	28 (4.97)		34 (6.00)	29 (4.92)	46 (6.96)		45 (7.45)	26 (4.24)	38 (6.33)	
Current, <5 cigarettes/d	19 (3.03)	12 (1.92)	22 (3.91)		10 (1.76)	14 (2.38)	29 (4.39)		29 (4.80)	14 (2.28)	10 (1.67)	
Current, ≥5 cigarettes/d	66 (10.51)	71 (11.34)	84 (14.92)		49 (8.64)	62 (10.53)	110 (16.64)		97 (16.06)	66 (10.77)	58 (9.67)	
Alcohol drinking (%)				0.654				0.003				<0.001
Never	535 (85.06)	529 (84.50)	459 (81.53)		490 (86.27)	507 (86.08)	526 (79.58)		465 (76.86)	524 (85.48)	534 (89.00)	
Abstinence	41 (6.52)	40 (6.39)	41 (7.28)		39 (6.87)	32 (5.43)	51 (7.72)		49 (8.10)	41 (6.69)	32 (5.33)	
<40 g/week	48 (7.63)	49 (7.83)	54 (9.59)		37 (6.51)	44 (7.47)	70 (10.59)		76 (12.56)	44 (7.18)	31 (5.17)	
≥40 g/week	5 (0.79)	8 (1.28)	9 (1.60)		2 (0.35)	6 (1.02)	14 (2.12)		15 (2.48)	4 (0.65)	3 (0.50)	
Physical activity (%)				0.021				0.003				0.009
Light	392 (62.42)	378 (60.48)	344 (61.21)		321 (56.51)	350 (59.63)	443 (67.12)		382 (63.25)	376 (61.44)	356 (59.43)	
Moderate	153 (24.36)	148 (23.68)	162 (28.83)		164 (28.87)	154 (26.24)	145 (21.97)		164 (27.15)	156 (25.49)	143 (23.87)	
Heavy	83 (13.22)	99 (15.84)	56 (9.96)		83 (14.61)	83 (14.14)	72 (10.91)		58 (9.60)	80 (13.07)	100 (16.69)	
Altitude ^2^ (%)				0.050				0.003				<0.001
High altitude	372 (77.50)	443 (83.58)	384 (80.67)		407 (76.07)	436 (82.58)	356 (84.16)		437 (87.75)	395 (79.64)	367 (74.59)	
Very high altitude	108 (22.50)	87 (16.42)	92 (19.33)		128 (23.93)	92 (17.42)	67 (15.84)		61 (12.25)	101 (20.36)	125 (25.41)	

^1^ The first survey result from 522 participants who participated twice was used here. Data are presented as the mean ± SD for continuous measures and as *n* (%) for categorical measures. ^2^ Altitude was determined by the participant’s annual residence time in a pasturing area. High altitude: lived in pasturing areas less than or equal to 6 months per year. Very high altitude: lived in pasturing area for more than 6 months per year.

**Table 3:** Associations between tertiles of three DP scores with MetS and its components among Tibetans in China (*n* = 2354 person-time) ^1^.

	Modern Pattern				Urban Pattern				Pastoral Pattern			
	T1	T2	T3	*p_trend_*	T1	T2	T3	*p_trend_*	T1	T2	T3	*p_trend_*
Median	−0.66	−0.24	0.52		−0.92	−0.14	0.98		−0.85	−0.04	0.76	
MetS												
Crude	1.00	0.68 (0.46, 1.01)	0.69 (0.46, 1.03)	0.102	1.00	0.97 (0.65, 1.43)	1.37 (0.92, 2.05)	0.098	1.00	1.74 (1.18, 2.59)	1.30 (0.87, 1.93)	0.212
Model 1	1.00	0.83 (0.57, 1.20)	1.00 (0.69, 1.46)	0.856	1.00	1.30 (0.88, 1.92)	2.68 (1.76, 4.10)	<0.001	1.00	1.20 (0.82, 1.75)	0.87 (0.59, 1.28)	0.442
Model 2	1.00	0.82 (0.56, 1.20)	0.94 (0.64, 1.39)	0.851	1.00	1.24 (0.83, 1.84)	2.40 (1.53, 3.76)	<0.001	1.00	1.20 (0.81, 1.78)	0.91 (0.61, 1.37)	0.610
Model 3	1.00	0.79 (0.51, 1.21)	0.92 (0.59, 1.44)	0.861	1.00	1.20 (0.79, 1.83)	2.25 (1.36, 3.73)	0.002	1.00	1.19 (0.77, 1.83)	1.02 (0.66, 1.60)	0.941
Model 4	1.00	0.69 (0.37, 1.29)	0.67 (0.35, 1.29)	0.257	1.00	1.52 (0.82, 2.80)	3.42 (1.65, 7.10)	0.001	1.00	1.52 (0.82, 2.82)	1.15 (0.60, 2.22)	0.710
Central obesity												
Crude	1.00	0.68 (0.45, 1.04)	0.75 (0.50, 1.14)	0.274	1.00	0.74 (0.48, 1.12)	0.54 (0.35, 0.82)	0.005	1.00	1.57 (1.03, 2.39)	1.10 (0.72, 1.68)	0.662
Model 1	1.00	0.77 (0.49, 1.21)	1.12 (0.71, 1.76)	0.451	1.00	1.02 (0.65, 1.59)	1.13 (0.72, 1.77)	0.584	1.00	0.89 (0.57, 1.40)	0.57 (0.36, 0.90)	0.016
Model 2	1.00	0.81 (0.52, 1.27)	1.18 (0.75, 1.87)	0.359	1.00	1.03 (0.66, 1.61)	1.13 (0.70, 1.83)	0.602	1.00	0.86 (0.54, 1.35)	0.57 (0.35, 0.92)	0.019
Model 3	1.00	0.73 (0.44, 1.19)	1.16 (0.70, 1.93)	0.393	1.00	0.99 (0.62, 1.57)	1.40 (0.81, 2.38)	0.216	1.00	1.13 (0.69, 1.85)	0.70 (0.42, 1.16)	0.150
Model 4	1.00	0.64 (0.30, 1.36)	1.26 (0.56, 2.81)	0.457	1.00	0.93 (0.45, 1.92)	1.95 (0.84, 4.51)	0.111	1.00	1.19 (0.55, 2.61)	0.83 (0.37, 1.88)	0.629
Elevated BP												
Crude	1.00	0.84 (0.57, 1.22)	1.34 (0.92, 1.96)	0.069	1.00	0.77 (0.53, 1.11)	0.79 (0.55, 1.14)	0.247	1.00	1.25 (0.87, 1.81)	1.03 (0.71, 1.50)	0.870
Model 1	1.00	1.06 (0.73, 1.55)	2.20 (1.49, 3.25)	<0.001	1.00	1.01 (0.70, 1.45)	1.63 (1.11, 2.38)	0.008	1.00	0.81 (0.56, 1.17)	0.62 (0.43, 0.91)	0.015
Model 2	1.00	0.97 (0.66, 1.43)	1.94 (1.29, 2.90)	0.001	1.00	0.91 (0.62, 1.33)	1.28 (0.85, 1.94)	0.195	1.00	0.82 (0.56, 1.22)	0.64 (0.43, 0.96)	0.030
Model 3	1.00	0.95 (0.60, 1.50)	2.07 (1.27, 3.39)	0.002	1.00	0.81 (0.52, 1.25)	1.08 (0.66, 1.76)	0.757	1.00	0.94 (0.59, 1.49)	0.73 (0.45, 1.18)	0.197
Model 4	1.00	0.99 (0.51, 1.92)	1.66 (0.82, 3.35)	0.137	1.00	0.75 (0.40, 1.42)	0.77 (0.37, 1.58)	0.483	1.00	1.06 (0.54, 2.07)	0.96 (0.48, 1.90)	0.887
Low HDL-C												
Crude	1.00	0.78 (0.64, 0.95)	0.61 (0.50, 0.75)	<0.001	1.00	1.28 (1.05, 1.56)	3.07 (2.50, 3.77)	<0.001	1.00	1.12 (0.92, 1.37)	1.04 (0.85, 1.27)	0.683
Model 1	1.00	0.77 (0.63, 0.94)	0.61 (0.49, 0.75)	<0.001	1.00	1.35 (1.10, 1.66)	3.41 (2.76, 4.23)	<0.001	1.00	1.08 (0.89, 1.33)	1.01 (0.83, 1.23)	0.947
Model 2	1.00	0.79 (0.64, 0.98)	0.58 (0.47, 0.72)	<0.001	1.00	1.30 (1.05, 1.61)	2.88 (2.27, 3.64)	<0.001	1.00	1.15 (0.92, 1.42)	1.06 (0.86, 1.32)	0.602
Model 3	1.00	0.88 (0.70, 1.10)	0.71 (0.56, 0.90)	0.005	1.00	1.31 (1.04, 1.63)	2.36 (1.83, 3.04)	<0.001	1.00	1.00 (0.79, 1.26)	0.97 (0.76, 1.23)	0.806
Model 4	1.00	0.82 (0.58, 1.17)	0.60 (0.41, 0.87)	0.007	1.00	1.42 (1.00, 2.01)	2.47 (1.66, 3.69)	<0.001	1.00	1.10 (0.76, 1.58)	0.85 (0.58, 1.23)	0.358
Elevated TAG												
Crude	1.00	0.84 (0.58, 1.22)	1.14 (0.79, 1.65)	0.349	1.00	0.83 (0.57, 1.20)	0.58 (0.39, 0.86)	0.007	1.00	0.93 (0.65, 1.35)	0.90 (0.62, 1.30)	0.565
Model 1	1.00	0.93 (0.63, 1.38)	1.50 (1.02, 2.22)	0.021	1.00	0.92 (0.63, 1.35)	0.79 (0.53, 1.19)	0.260	1.00	0.76 (0.51, 1.12)	0.72 (0.48, 1.08)	0.113
Model 2	1.00	0.94 (0.63, 1.40)	1.50 (1.00, 2.26)	0.033	1.00	0.87 (0.58, 1.28)	0.73 (0.46, 1.14)	0.163	1.00	0.73 (0.48, 1.09)	0.69 (0.46, 1.05)	0.088
Model 3	1.00	0.93 (0.71, 1.22)	1.19 (0.89, 1.58)	0.179	1.00	0.88 (0.68, 1.15)	0.97 (0.72, 1.30)	0.821	1.00	0.83 (0.62, 1.10)	0.81 (0.61, 1.07)	0.141
Model 4	1.00	0.64 (0.31, 1.34)	0.76 (0.36, 1.60)	0.533	1.00	0.72 (0.36, 1.45)	0.85 (0.38, 1.88)	0.689	1.00	0.69 (0.32, 1.48)	0.95 (0.44, 2.03)	0.915
Impaired FBG												
Crude	1.00	0.88 (0.60, 1.29)	1.05 (0.71, 1.54)	0.697	1.00	0.76 (0.51, 1.13)	0.32 (0.19, 0.52)	<0.001	1.00	1.19 (0.82, 1.76)	1.66 (1.14, 2.43)	0.008
Model 1	1.00	0.97 (0.65, 1.46)	1.43 (0.94, 2.16)	0.074	1.00	0.89 (0.59, 1.33)	0.44 (0.27, 0.73)	0.001	1.00	0.95 (0.63, 1.43)	1.32 (0.88, 1.97)	0.162
Model 2	1.00	0.95 (0.63, 1.43)	1.48 (0.96, 2.29)	0.054	1.00	0.95 (0.63, 1.43)	0.49 (0.29, 0.81)	0.005	1.00	1.00 (0.65, 1.52)	1.38 (0.91, 2.09)	0.113
Model 3	1.00	0.94 (0.71, 1.24)	1.16 (0.87, 1.56)	0.245	1.00	0.95 (0.70, 1.23)	0.74 (0.54, 1.02)	0.065	1.00	1.05 (0.78, 1.41)	1.42 (1.06, 1.90)	0.015
Model 4	1.00	1.24 (0.62, 2.47)	1.18 (0.57, 2.45)	0.689	1.00	1.06 (0.55, 2.04)	0.79 (0.36, 1.73)	0.564	1.00	1.30 (0.63, 2.67)	1.68 (0.80, 3.54)	0.166

^1^ Crude was unadjusted model. Model 1 was adjusted for age and sex; Model 2 was further adjusted for marital status, education, insurance status, household income, smoking status, alcohol consumption, and physical activity; Model 3 was further adjusted altitude; Model 4 was the same as Model 3 but only included those who has both baseline and follow-up data. DP scores were equally divided into thirds. T means tertile (T1–T3). Tibetan adults who attended at least one wave were included in 2018 and 2022 (*n* = 2354 person-time).

## Data Availability

The data can be obtained by contacting the corresponding author.

## Supplementary Materials

The following supporting information can be downloaded at: https://www.mdpi.com/article/10.3390/nu15092226/s1, Figure S1: Scree plot; Figure S2: Directed acyclic graph for potential confounders selection between major DPs with MetS; Table S1: Demographic and lifestyle characteristics of subjects in urbanized and semi-urbanized settled Tibetan communities (*n* = 1832); Table S2: Associations between tertiles of three DPs scores with MetS and its components by gender among Tibetans in China; Table S3: Subgroup analyses of the associations between tertiles of major DPs scores and MetS and its components (*n* = 522).
